# Chromosomal disorders: estimating baseline birth prevalence and pregnancy outcomes worldwide

**DOI:** 10.1007/s12687-017-0336-2

**Published:** 2017-09-26

**Authors:** Sowmiya Moorthie, Hannah Blencowe, Matthew W. Darlison, Stephen Gibbons, Joy E. Lawn, Pierpaolo Mastroiacovo, Joan K. Morris, Bernadette Modell, Congenital Disorders Expert Group, A. H. Bittles, H. Blencowe, A. Christianson, S. Cousens, M. W. Darlison, S. Gibbons, H. Hamamy, B. Khoshnood, C. P. Howson, J. E. Lawn, P. Mastroiacovo, B. Modell, S. Moorthie, J. K. Morris, P. A. Mossey, A. J. Neville, M. Petrou, S. Povey, J. Rankin, L. Schuler-Faccini, C. Wren, K. A. Yunis

**Affiliations:** 1 PHG Foundation, 2 Worts Causeway, Cambridge, UK; 2 Centre for Maternal, Adolescent, Reproductive, and Child Health, London School of Hygiene and Tropical Medicine, London, UK; 3 WHO Collaborating Centre for Community Genetics, Centre for Health Informatics and Multiprofessional Education (CHIME), University College London, London, UK; 4 Department of Geography and Environment, London School of Economics, London, UK; 5 Coordinating Centre of the International Clearinghouse for Birth Defects Surveillance and Research, Rome, Italy; 6 Centre for Environmental and Preventive Medicine, Wolfson Institute of Preventive Medicine, Barts and the London School of Medicine and Dentistry, Queen Mary University of London, London, UK

**Keywords:** Chromosomal disorders, Birth prevalence, Mortality, Disability

## Abstract

Chromosomal disorders, of which Down syndrome is the most common, can cause multi-domain disability. In addition, compared to the general population, there is a higher frequency of death before the age of five. In many settings, large gaps in data availability have hampered policy-making, programme priorities and resource allocation for these important conditions. We have developed methods, which overcome this lack of data and allow estimation of the burden of affected pregnancies and their outcomes in different settings worldwide. For example, the methods include a simple equation relating the percentage of mothers 35 and over to Down syndrome birth prevalence. The results obtained provide a starting point for consideration of services that can be implemented for the care and prevention of these disorders.

**Electronic supplementary material:**

The online version of this article (10.1007/s12687-017-0336-2) contains supplementary material, which is available to authorized users.

## Introduction

Chromosomal disorders are caused by changes occurring in either chromosome number or structure usually during the formation of gametes or soon after fertilisation. These changes can affect the autosomes or the sex chromosomes (XX in females, XY in males), with chromosomal disorders divided into the two corresponding groups. Most autosomal disorders cause death before the age of 5 years or multi-domain disability. However, the availability of appropriate medical care in high-income settings means that there are fewer deaths in those with Down syndrome before the age of five (Glasson et al. 2016; Kucik et al. 2013; Wu and Morris 2013). Disorders of the sex chromosomes have a lesser effect on survival but can cause infertility, congenital malformations such as congenital heart disease in Turner syndrome (Pinsker 2012), as well as having neurodevelopmental and psychological impacts (Bojesen and Gravholt 2007; Pinsker 2012; Tartaglia et al. 2015) (Table 1). Chromosomal disorders can occur in any pregnancy (most arise sporadically), but the risk of having a pregnancy affected by Down syndrome and trisomies T13 and T18 is known to increase with maternal age (Hassold et al. 1996; Hassold and Hunt 2009; Hook et al. 1983).

**Table 1:** Chromosomal disorders included in MGDb. A small minority of those with chromosomal disorders is mosaics (have both normal and abnormal cells) and this causes a less severe disorder. In MGDb, Edwards syndrome (+18) and Patau syndrome (+13) are treated together because their outcomes are very similar. Rare autosomal disorders include triploidy, other trisomies, unbalanced structural rearrangements, markers, and microdeletions

Group	Disorder	Maternal age-related?	% of cases that have associated malformations	Other clinical features	Source of prevalence data
Autosomal disorders	Down syndrome (T21)	Yes	40–50	Learning difficulties, reduced immunity, premature ageing	Hecht and Hook (1996), Morris et al. (2014)
	Edwards syndrome (T18)	Yes	100	Very severe developmental disorders resulting in early death in infancy	Savva et al. (2010)
	Patau syndrome (T13)				
	Rare autosomal disorders	Some	Most	Diverse group, severe learning difficulties, other problems	Wellesley et al. (2012)
Sex chromosome disorders	Turner syndrome (45,X)	No	30	Failure of puberty, sterility	Alberman and Creasy (1977), EUROCAT
	Klinefelter syndrome (XXY)	Some	–	Hypogonadism, sterility	Bojesen et al. (2003)

The Modell Global Database of Congenital Disorders (MGDb) uses a set of defined methods to relate demographic data to the known birth prevalence of selected congenital disorders, to generate estimates relevant to public health, policy making and clinical practice (Blencowe et al. 2016; Moorthie et al. 2016). This paper, the fourth in a supplement on estimation of congenital disorders, describes the methods used in MGDb to estimate the baseline birth prevalence and pregnancy outcomes of the chromosomal disorders listed in Table 1. Balanced structural rearrangements and sex chromosome disorders that cause minimal health problems (e.g. XYY and XXX) are not included. This paper will be of interest to those seeking to estimate the burden of chromosomal disorders and potential effect of interventions in settings with limited data including policy and programme planners.

### Overview of methodology

The incidence of most congenital disorders, including Down syndrome, is usually expressed as birth prevalence. This is for practical purposes, as it is recognised that many affected embryos fail to implant or miscarry in early pregnancy, hence never come to the attention of health services. We too have followed this convention and equated birth prevalence with incidence. The objective of MGDb is to estimate numbers of births affected by one or more congenital disorders and outcomes (live birth, stillbirth or termination of pregnancy (TOP)) in the no-care situation and with current care. The starting point for this is baseline prevalence (i.e. live birth and stillbirth) in the absence of care. MGDb provides epidemiological estimates relating to birth prevalence, pregnancy outcomes, survival and effect of interventions. These estimates are not definitive and require refinement, but can be used by those working in public health or by policy makers as a starting point to assess service needs and policy gaps in settings with little or no data.

To estimate the potential live birth prevalence of chromosomal disorders for different populations, we used a step-wise approach:

- Step 1: Estimation of the expected prevalence of Down syndrome among live births in the absence of any intervention using the established relationship with maternal age
- Step 2: Estimation of the birth prevalence of other chromosomal disorders and foetal deaths
- Step 3: Estimation of the overall baseline birth prevalence of chromosomal disorders in the absence of any intervention
- Step 4: Estimation of actual birth prevalence based on the availability and estimated access to prenatal diagnosis and termination of pregnancy (TOP) for foetal impairment
- Step 5: Comparisons of estimates with registry data

## Step 1: Expected prevalence of Down syndrome among live births in the absence of intervention

### Maternal age-related rates for live births with Down syndrome

The live birth prevalence of chromosomal disorders in the absence of intervention was originally described in populations mainly of northern European origin (Hecht and Hook 1996; Hook and Hamerton 1977). These studies showed that the birth prevalence of Down syndrome and trisomies T13 and T18 is related to maternal age. Klinefelter syndrome was also shown to have a weak maternal age relationship, while the birth prevalence of Turner syndrome (45,X) and most other chromosomal disorders are approximately constant across these populations.

Studies have described ethnic differences in Down syndrome birth prevalence; however, it is uncertain if these differences are due to underlying biological factors or as a result of differential use of screening services or due to differential ascertainment (Bishop et al. 1997; Forrester and Merz 2003; Khoshnood et al. 2000). In the absence of studies that provide evidence to support significant differences in these rates between population groups, or any effect of environmental or other genetic factors, we have assumed that the observations relating to risk in the initial studies are generalizable to all populations globally.

**Fig. 1:**
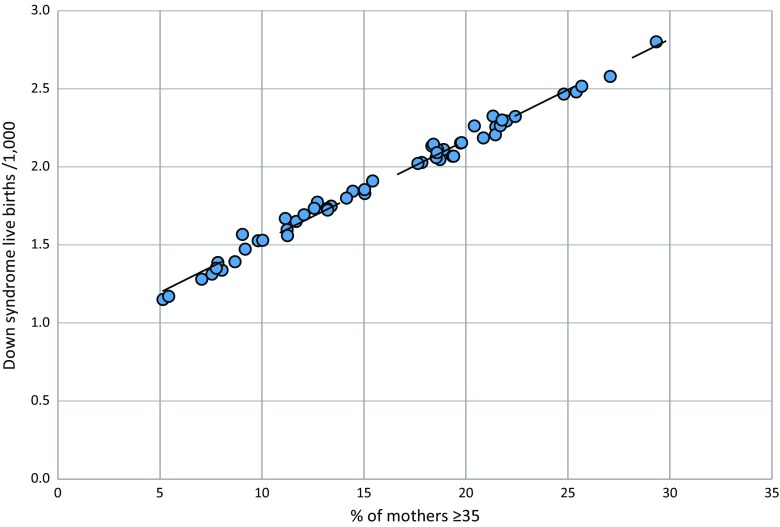
Relationship between percentage of mothers ≥ 35 plus and Down syndrome potential live birth prevalence. Live birth prevalence was estimated using the full range of risks in Table 2 for 54 high-income and eastern European countries with reliable demographic data. The selection covers a wide spread of rates because the proportion of older mothers is high in high-income countries but low in eastern Europe. Calculations are for 2005–2009, but results are similar for any time interval. Coefficient of correlation = 0.9944

The relationship between maternal age and risk of a Down syndrome-affected live birth is employed in counselling pregnant women in relation to their risk of an affected birth (Bray et al. 1998; Cuckle et al. 1987; Hecht and Hook 1996). However, the data used to construct the initial curve were collected between 1966 and 1980, when the proportion of older mothers was at its lowest in the relevant populations, and confidence intervals for the oldest groups were consequently very wide. Subsequent studies have included more data points (Ferguson-Smith and Yates 1984; Morris et al. 2002, 2005). We therefore combined the data of Hecht and Hook (1996) and Morris et al. (2002) in order to maximise the number of older mothers included (see ESM 1: Table 1 online resources). We used these data to produce a curve (ESM 1: Fig. 1 online resources) to estimate Down syndrome risk by 5-year age groups (Table 2). Five-year age groups were employed in order to relate to demographic data which are presented using these age intervals. The resulting rates were very close to those reported by Wu and Morris (2013) and were used to develop an equation-based method to estimate Down syndrome live birth prevalence.

**Table 2:** Risk of a Down syndrome live birth by 5-year maternal age intervals. (based on Hecht and Hook (1996), Morris et al. (2002))

Maternal age group	Down syndrome live births	Total live births	Down syndrome live births/1000	Lower 95% CI	Upper 95% CI
15–19	71	124,562	0.61	0.54	0.67
20–24	387	541,511	0.71	0.67	0.75
25–29	533	585,770	0.87	0.83	0.90
30–34	390	260,378	1.46	1.40	1.51
35–39	380	84,373	4.58	4.42	4.74
40–44	276	17,655	15.71	14.98	16.44
45–49	46	893	33.50	28.54	38.46
Total	2085	1,615,142	1.52		
Total 15–34	1381	1,5122,21	0.913		

### Relating risk to maternal age distribution

**Fig. 2:**
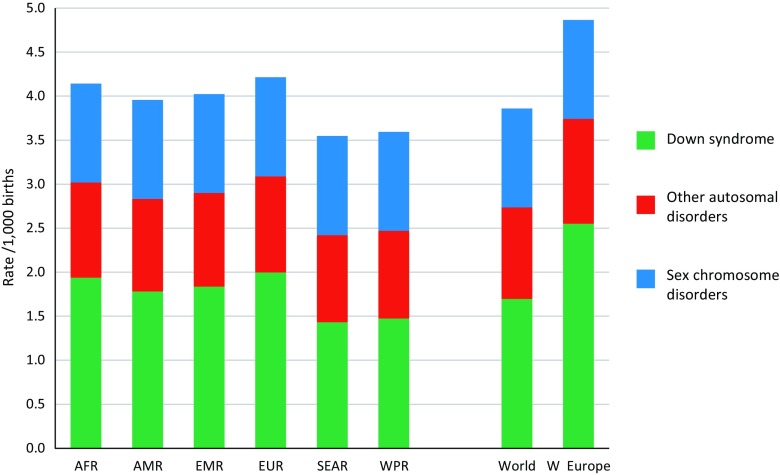
Estimated baseline total, birth prevalence of chromosomal disorders by WHO region in 2010–2014. AFR: African, AMR: American, EMR: Eastern Mediterranean, SEAR: South-East Asian, WPR: Western Pacific Region, W. Europe: Western Europe

The proportion of all births to older women changes with population age distribution, social changes and utilisation of family planning (ESM 1: Fig. 2 online resources). Therefore, the live birth prevalence of maternal age-related disorders differs between populations and over time. In theory, Down syndrome live birth prevalence can be estimated for every country by applying the risks in Table 2 to the UNWPP estimates of maternal age distribution. However, there are possible sources of error in this approach.

Inaccuracy in age reporting is common, particularly in lower-income settings, among less educated and older groups and among women. This can lead to ‘age heaping’, which is the tendency to estimate age up to the nearest figures ending in 0 or 5 (UN Demographic Yearbook special issue on age heaping, 2017). Age heaping can be quantified using Whipple’s index, which provides a means of assessing the reliability of age data (Shryock et al. 1976). Index scores range between 100 and 500; a high score (generally above 125) indicates lower quality data. A review of Whipple’s index for 1985 to 2003 in the United Nations Demographic Yearbook (UNDY) special issue on age heaping (UN Demographic Yearbook special issue on age heaping 2017) shows that only population age data from the European and Western Pacific regions are truly reliable, as these index scores are close to 100 (online resources ESM 1: Table 2). This is likely to apply even more to maternal age data, especially for older age groups, as age heaping is most marked in the oldest age group. Therefore, only maternal age data for populations with a Whipple’s index of 105–110 can be used to support accurate calculation of Down syndrome birth prevalence using the 5-year maternal age groups in Table 2.

If age heaping is not taken into account, it can lead to inaccuracies in the estimation process and overestimation of the live birth prevalence of Down syndrome. We sought to reduce the effect of heaping by developing a formula to describe the relationship between the percentage of mothers aged ≥ 35 and Down syndrome live births. This was undertaken in two steps. Firstly, we used the rates for Down syndrome birth prevalence in Table 2 along with country-specific age distribution to estimate country-specific birth prevalence by five-year intervals. This was done only using age distribution data from countries with little age heaping, as assessed by their Whipple’s index. Fifty-four countries had evidence of reliable demographic age data. These data included countries from western Europe with the highest estimated proportion of mothers ≥ 35 and eastern Europe where the proportion of mothers ≥ 35 is the lowest globally. We then plotted the calculated Down syndrome birth prevalence by country against total WPP births to mothers’ ≥ 35, in these countries, the rationale being that the larger ≥ 35 age group can easily accommodate inaccuracies in the reporting of maternal age at the oldest groups. Figure 1 shows a linear relationship between the percentage of mothers aged ≥ 35 and Down syndrome live birth prevalence. This empirical relationship can be described mathematically by the following formula:

$$ y= mx+b $$

where:

*y*: down syndrome potential live births/1000
*m*: the slope of the line (0.067)
*x*: percentage of mothers ≥ 35 × 0.067
*b*: the intercept (0.834)

A comparison of estimated potential live birth prevalence, applying the risks to the five-year bands, compared to the formula above using WPP 2015 maternal age data from 1950 to 2015, can be found in the online resources for three countries (Online Figs. 3, 4 and 5)—United Kingdom, Nigeria and Iran. Identical results are obtained for the UK using both methods, which has a low Whipple’s index. However, the former approach produces higher estimates for countries with a high Whipple’s index (Iran and Nigeria). To avoid overestimation and for the sake of consistency, we use the formula above to estimate Down syndrome potential live birth prevalence in any population, including those for which maternal age data are highly reliable. Maternal age distribution data were obtained from the United Nations World Populations Prospects - UNWPP (United Nations Population Division 2015)

## Step 2: Estimating potential live birth rates of other chromosomal disorders and foetal deaths

In MGDb, estimates for T13 and T18 are grouped together as they have similar outcomes. The birth prevalence of these disorders is also maternal age-related, with a joint live birth prevalence approximately 19.5% of that of Down syndrome (Savva et al. 2010). We used this figure to estimate the combined potential live birth prevalence of these two conditions.

Rates for rare chromosomal disorders are also based on data available in the literature. Using EUROCAT data, Wellesley et al. (2012) found a total birth prevalence of 0.74/1000 for ‘rare chromosomal disorders’. However, the study indicates a likely 25% foetal death rate had prenatal diagnosis not been available. Taking this into considerations gives a baseline live birth prevalence of around 0.55/1000.

The baseline live birth prevalence of Turner syndrome (45,X) used in MGDb was derived from an average of country-specific rates using publicly accessible EUROCAT data for the years 2000–2009. Data included in the analysis was from registries that submit raw data and from countries where TOP is legal and reported (Ireland, Malta and Poland were excluded). Average EUROCAT data indicate that the potential live birth prevalence of Turner syndrome (45,X) is approximately 0.175/1000.

The birth prevalence of Klinefelter syndrome (XXY) is weakly related to maternal age because around half of the responsible non-disjunctions are of paternal origin (Bojesen et al. 2003; Carothers and Filippi 1988; Ferguson-Smith and Yates 1984). However, we have not used a maternal age relationship to estimate birth prevalence in MGDb, but used the average potential live birth prevalence of 0.703/1000 reported by Morris et al. (2008)

### Estimating foetal death rates

Most publications report live birth prevalence of chromosomal disorders because they aim to provide precise risks for genetic counselling. However, to assess their public health significance, it is necessary to include stillbirths and to allow for the effects of prenatal diagnosis in countries where pregnancy termination is legal. We use foetal death, defined as losses *in utero* after 20 weeks as a proxy for stillbirths. To obtain potential total birth prevalence, estimates for foetal deaths were added to the estimated live birth rates obtained using the formula.

Rates for pregnancy losses have been reported and shown to differ with stage of gestation and the condition (Morris and Savva 2008; Morris and Wald 1999). In MGDb, we use rates for foetal deaths i.e. from 20 weeks of pregnancy onwards. There are differences in foetal death estimates, and this is particularly marked for Down syndrome, such as 5% by Halliday et al. (1995) and 12% by Morris and Wald (1999). We have used the conservative figure of 5% of total births (equivalent to 5.3% of live births) until more data are available. Table 3 summarises potential foetal death rates in the absence of any intervention, by main disorder group along with sources of data.

**Table 3:** Potential percentage foetal deaths in the absence of any intervention by chromosomal disorder

Diagnosis	Potential live births/1000	Foetal deaths, % of live births	Foetal deaths, % of total births^a^	Source used for foetal death rate
Down syndrome	Estimated using maternal age	5.3	5	Morris et al. (1999)
Other trisomies	Estimated as 19.5% of Down syndrome prevalence	122	55	Morris et al. (2008)
Other autosomal disorders	0.55	28	25	Wellesley et al. (2012)
Turner syndrome	0.175	27	21	EUROCAT average^b^
Klinefelter syndrome	0.703	3.0	2.9	EUROCAT average^b^

Note: When only information on live birth prevalence is available, the figures in the third column can be used to calculate foetal deaths. When only information on total birth prevalence is available, the figures in the fourth column can be used to calculate foetal deaths

^a^Total births = live births plus stillbirths

^b^Data source: EUROCAT website. Data from the registries that submit raw, unaggregated data were included in the analysis. Includes only data from countries where TOP is legal and reported (Ireland, Malta and Poland were excluded)

These calculations can be used to produce baseline birth prevalences for any population. Although a large proportion of miscarriages (losses before 20 weeks) are associated with chromosomal aneuploidy and risk of miscarriage accordingly rises with maternal age (Alberman and Creasy 1977), miscarriage is not considered here. Figure 2 shows estimates of baseline birth prevalence of chromosomal disorders by WHO region for 2010, calculated using this method.

## Step 3: Estimation of the overall baseline birth prevalence of chromosomal disorders

The overall prevalence is calculated by summing the baseline birth prevalence, including both live births and foetal deaths of all included chromosomal disorders from steps 1 and 2. This approach has the advantage of providing a starting point from which the impact of interventions can be examined and evaluated.

## Step 4: Estimation of actual birth prevalence

The results from step 3 apply directly for countries where termination of pregnancy for foetal impairment is illegal, but must be adjusted to allow for the effects of prenatal diagnosis in countries where termination is legal.

### Adjusting for factors that impact on pregnancy outcomes

Most countries with appropriate resources where TOP for foetal impairment is legal have developed antenatal screening programmes specifically targeted at Down syndrome. These programmes identify pregnancies with an increased likelihood of foetal anomalies and offer an option for early diagnosis. Although targeted specifically at Down syndrome, antenatal screening and diagnosis programmes are likely to identify other chromosomal anomalies, including trisomies 13 and 18 and 45,X. The identification of individuals with other chromosomal anomalies is usually made later in life, following other diagnostic investigations. Prenatal diagnosis may allow planning and preparation for an affected family or, in places where it is legal and acceptable, may lead to a choice of pregnancy termination.

Techniques and policies for prenatal diagnosis of chromosomal disorders have evolved greatly over the last half-century impacting on the proportion of cases detected and their acceptability to pregnant women and their partners. For example, the development of combined screening has led to a policy in many countries of offering risk information and screening to all pregnant women, followed by definitive diagnosis to those who have been identified as at high risk.

Umbrella registries (ICBDSR and EUROCAT) have recorded almost the entire evolution of the population impact of screening and diagnosis for chromosomal disorders in high-income settings. Consequently, country-specific data on termination of pregnancy from umbrella registries are used for these estimates when available. Otherwise, we have used publicly available data from EUROCAT to estimate the average proportions of pregnancies terminated by diagnosis by 5-year interval, in participating countries where termination of pregnancy is legal and reported (Table 4). Information relating to legality of termination of pregnancy (TOP) for fetal impairment was obtained from the UN (United Nations 2014).

**Table 4:** Average percentage rates for termination of pregnancy for chromosomal disorders for 16 countries where termination for foetal impairment is legal and reported. Data source: EUROCAT website. Data from the registries that submit raw, unaggregated data were included in the analysis. Includes only data from countries where TOP is legal and reported (Ireland, Malta and Poland were excluded)

Disorder group	1980–1984	1985–1989	1990–1994	1995–1999	2000–2004	2005–2009
Down syndrome	4.3	14.7	28.3	45.2	52.9	58.7
Other trisomies	8.2	39.8	57.1	67.7	75.7	81.4
Rare disorders	9.3	23.7	54.6	47.8	41.7	42.1
Turner syndrome	32.9	45.1	58.2	64.9	65.7	69.9

We compared these rates with those of the UK National Down Syndrome Cytogenetic Register (National Down Syndrome Cytogenetic Register (NDSCR) (2017)) in order to examine the degree to which they reflect the informed choices of pregnant women. NDSCR data for England and Wales in 2009 show that the great majority of women are informed of the availability of testing in time for the option of prenatal diagnosis; around 70% request screening for chromosomal disorders and over 95% of those with a definitive diagnosis of a severe disorder choose to terminate the pregnancy (Springett and Morris 2010; Springett et al. 2014). The resulting 60% termination rate calculated from NDSCR data is close to the EUROCAT average (58.7%).

Due to the absence of readily accessible observational data on the availability and utilisation of prenatal diagnosis, we have assumed that it will be available in those countries where TOP is legal and access to care will impact utilisation. In MGDb, we have used the EUROCAT average rates for settings that have a policy of universal screening. In settings where termination of pregnancy is legal but there is no evidence of a universal screening policy and no available data on termination rates, we have assumed testing is offered only to high-risk groups. In these settings, we have used a rate which is 50% of the EUROCAT average. We have assumed that all women worldwide with access to screening and TOP will act in the same way in terms of uptake of this service. It is assumed that no widespread screening for chromosomal disorders or terminations takes place in countries where termination of pregnancy for foetal impairment is illegal. This is likely to lead to slight overestimation of actual live birth prevalence, as a minority of couples can usually access such services, for example as in the case of Brazil (Diniz and Medeiros 2010; Horovitz et al. 2013).

## Step 5: Comparison of estimates with registry data for Down syndrome

**Fig. 3:**
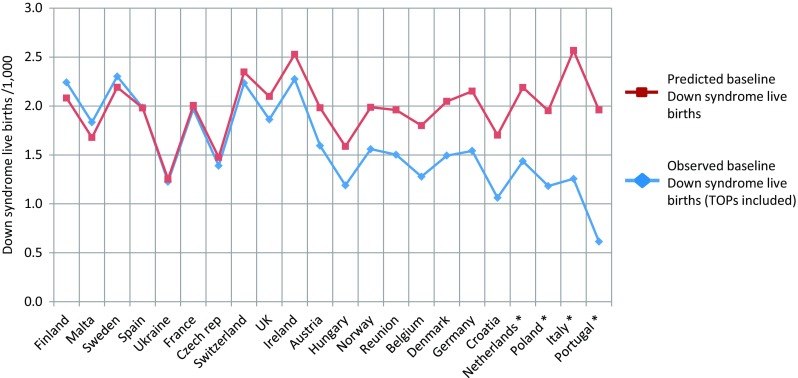
Comparison of estimated potential Down syndrome live births/1000 with observed potential live births calculated from EUROCAT registry data for 2000–2009. In order to make the comparison, EUROCAT total Down syndrome birth prevalence was converted to potential live birth prevalence, by deducting 5% to allow for potential foetal deaths, and deducting 30% from reported terminations to allow for spontaneous losses had these pregnancies continued. Countries ranked in descending order of discrepancy. *Countries with the widest discrepancy

**Fig. 4:**
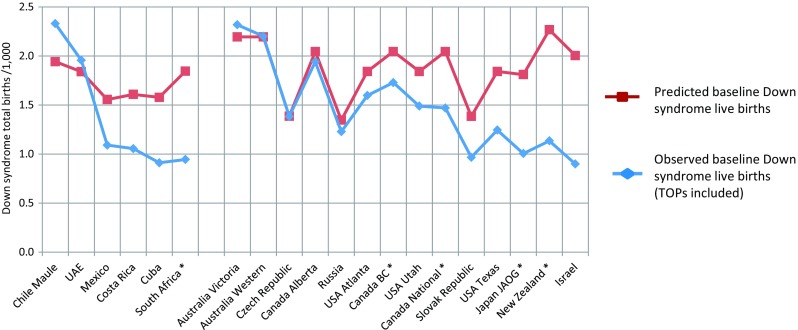
Comparison of estimated potential Down syndrome live births/1000, with observed potential live births calculated from ICBDSR registry data for 2000–2005. The six left-hand registries report from lower-income settings, and the right hand registries report from higher-income settings. *Registers where termination for foetal impairment is legal but not reported

Direct comparisons between estimates and reported data from low-middle-income settings are difficult to interpret due to weaknesses in reported data. However, we can compare differences between estimated and reported baseline prevalence rates for countries contributing to EUROCAT (Fig. 3) and ICBDSR (Fig. 4). Rates are comparable for 10 of the 22 countries contributing to EUROCAT. For the remaining 12 countries, the observed rate is 20–50% lower than predicted. There is recognised under-ascertainment in the four countries with the widest difference (starred)—with under-ascertainment demonstrated in Italy, Netherlands (de Graaf et al. 2011) and Portugal (Leoncini et al. 2010) and inevitable for Poland because terminations are not reported. Similarly, there is good agreement for six international registries contributing to ICBDSR. Discrepancies are evident for countries not reporting TOP (South Africa, British Colombia, Japan and New Zealand). The lower estimated than reported rate for Chile suggests either unreliable maternal age data or over-diagnosis of Down syndrome. Wide discrepancies for Mexico, Costa Rica and Cuba suggest substantial under-ascertainment.

Based on the examples above, under-ascertainment would seem to explain much of the difference between estimated Down syndrome birth prevalence and reported rates from registry data in high-income settings. Under-ascertainment might be of births, foetal deaths, terminations of pregnancy or a combination of all three. Under-ascertainment of foetal death (current estimated total = 5% of continuing pregnancies) would not greatly affect the reported birth prevalence of Down syndrome. Under-ascertainment seems more likely for terminations than for live births, particularly where there is widespread use of private maternity services or termination of pregnancy is illegal or a highly politicised issue. Under-ascertainment of terminations for other trisomies has been documented in the USA (Crider et al. 2008). Selective under-ascertainment of terminations may be suspected when both (a) the reported total birth prevalence is substantially lower than predicted and (b) the reported proportion of terminations is substantially lower than expectation.

## Discussion

### Strengths of method

Large gaps in data availability regarding chromosomal disorders have hampered policy-making, programme priorities and resource allocation for these important conditions. The methods proposed above allow an estimate of the burden of affected pregnancies and their outcomes from all settings, even those with sparse data. These estimates provide a starting point for consideration of services that can be implemented to address the burden of these disorders. The strengths of the method lie in the fact that it provides a relatively simple process to estimate the birth prevalence and pregnancy outcomes of chromosomal disorders. The availability of estimates of maternal age distribution from UNWPP makes it possible to develop country-specific estimates of the baseline birth prevalence of Down syndrome and other maternal age-related chromosomal disorders, regardless of resource level. Estimates can be made for other chromosomal disorders using rates either from the literature or through analysis of data available from EUROCAT—which is an established surveillance network.

When data is already collected on birth prevalence of these conditions by population-based registries, the method outlined here provides an independent means to assess ascertainment. As mentioned previously, these methods do not allow definitive estimates, but provide order to magnitude estimates that can be used by public health and policy makers to assess burden of disease and evaluate service needs. The steps taken and assumptions made allow this method to be applied across global populations which is an advantage, and comparison with observational data shows that rates are comparable in many cases. Where there are discrepancies, these can, to a large degree, be explained by under-ascertainment. However, the assumptions made in this process can add towards uncertainty around the estimates, and these are discussed below.

### Limitations of model

Potential limitations of the estimation process include the use of five-year maternal age bands. Curves to estimate Down syndrome risk are usually constructed using one-year age bands. Given the strong relationship between maternal age and risk, this allows more accurate risk assessment especially for use in a counselling setting. In order to relate to demographic data, which is usually presented in 5-year bands, we have used 5-year age groups to relate risk to maternal age. This could introduce uncertainty at two levels. Firstly, we have amalgamated two data sets to produce a risk curve. The data of Hecht and Hook (1996) were collected between 1966 and 1990 whereas that of Morris et al.(2002) were for the time period 1990–1998. As the data are from different time periods, there is a possibility that differences in maternal age structure between these periods impact on calculated one-year risk. However, this will only be the case if there is temporal difference between the maternal age relationship, which we think is unlikely. Secondly, the risks within each 5-year age band are, in essence, an average across this wider age group, which is likely to be influenced by the exact distribution of risk across these age bands.

We have assumed that risks are constant over time and that there are no ethnic differences. Although studies have been published examining ethnic differences in prevalence of Down syndrome, it is unclear at this time whether this is as a result of underlying biological factors or as a result of differential health care utilisation or social factors.

Another potential limitation with the estimation process includes the assumption that historical high-income country setting rates apply to all countries with a lack of data, and where there is access to termination of pregnancy, women will choose in a manner comparable to those of their European counterparts. This can contribute to uncertainty, firstly, as although current surveillance systems in these countries are robust, individual registries can differ in ascertainment due to a number of factors (e.g. resources, extent and mode of access to records etc.) as well as at the proportion of the total population they cover. This can lead to differences between countries in reported rates, especially for outcomes that do not end in an affected birth such as foetal death/stillbirth and termination of pregnancy. Secondly, there is a high rate of TOP for Down syndrome, other autosomal disorders and Turner syndrome in high-income countries where termination is legal. This assumption may therefore overestimate TOPs in other settings and therefore underestimate the number of foetal deaths and live births. Termination may be under-reported from countries where termination for foetal impairment is legal, but the issue is highly politicised. The extent to which termination reflects, informed parental choice has not been sufficiently investigated. Estimation of termination of pregnancy is based on EUROCAT and ICBDSR registry data. However, there may be considerable under-ascertainment in some countries (Leoncini et al. 2010). When this is the case, live birth prevalence and mortality due to the disorder are overestimated.

Although it is desirable to have as accurate as possible estimates for public health and service planning purposes, often this must be balanced against the feasibility of obtaining such data. Our aim has been to create a simple method that can utilise available data to start assessing service needs for those with chromosomal disorders.

## Conclusion

Our aim is to describe a simple method to estimate the birth prevalence of chromosomal disorders summarised in Table 2. The formula-based method used for estimates of Down syndrome birth prevalence derived from the proportion of mothers’ ≥ 35 is a useful tool for public health purposes. The method can be applied for any country, at any time period, and can be employed to assess ascertainment in local registries. In fact, calculation using maternal age distribution has already been suggested for assessing ascertainment of chromosomal disorders in registries (Leoncini et al. 2010) and is used by EUROCAT as a data quality indicator (Loane et al. 2011, 2013).

## Electronic supplementary material

ESM 1 (DOCX 419 kb)

## Abbreviations

EUROCAT: European Surveillance of Congenital Anomalies
ICBDSR: International Clearinghouse for Birth Defects Surveillance and Research
MGDb: Modell Global Database of Congenital Disorders
TOP: Termination of pregnancy
UN WPP: United Nations World Population Prospects
