# Supplementary material for: Chromosomal disorders: estimating baseline birth prevalence and pregnancy outcomes worldwide
Source: J Community Genet. 2017 Sep 26;9(4):377–86. doi: 10.1007/s12687-017-0336-2 (PMC6167258; doi:10.1007/s12687-017-0336-2)
Supplement: Supplementary file 1 — (DOCX 419 kb) [file 12687_2017_336_MOESM1_ESM.docx]

**Online resources**

Table 1. Down syndrome live birth prevalence by single completed year of maternal age. Amalgamated data from Hecht and Hook 1996 and Morris et al. 2002.

|  | HECHT & HOOK 1996 | | |  |  | MORRIS ET AL. 2002 | |  | AMALGAMATED DATA | | |  |  |
| --- | --- | --- | --- | --- | --- | --- | --- | --- | --- | --- | --- | --- | --- |
| Mat age at birth | Total Livebirths | Down live births | Down live births /1,000 | lower 90% CI | upper 90% CI | Total live births | Down live births (if no TOP) | Down live births /1,000 | Total live births | Down live births | Down's /1,000 live births | Lower 95% CI | Upper 95% CI |
|  |  |  |  |  |  |  |  |  |  |  |  |  |  |
|  |  |  |  |  |  |  |  |  |  |  |  |  |  |
|  |  |  |  |  |  |  |  |  |  |  |  |  |  |
|  |  |  |  |  |  |  |  |  |  |  |  |  |  |
|  |  |  |  |  |  |  |  |  |  |  |  |  |  |
|  |  |  |  |  |  |  |  |  |  |  |  |  |  |
|  |  |  |  |  |  |  |  |  |  |  |  |  |  |
|  |  |  |  |  |  |  |  |  |  |  |  |  |  |
|  |  |  |  |  |  |  |  |  |  |  |  |  |  |
|  |  |  |  |  |  |  |  |  |  |  |  |  |  |
|  |  |  |  |  |  |  |  |  |  |  |  |  |  |
|  |  |  |  |  |  |  |  |  |  |  |  |  |  |
|  |  |  |  |  |  |  |  |  |  |  |  |  |  |
|  |  |  |  |  |  |  |  |  |  |  |  |  |  |
|  |  |  |  |  |  |  |  |  |  |  |  |  |  |
|  |  |  |  |  |  |  |  |  |  |  |  |  |  |
|  |  |  |  |  |  |  |  |  |  |  |  |  |  |
|  |  |  |  |  |  |  |  |  |  |  |  |  |  |
|  |  |  |  |  |  |  |  |  |  |  |  |  |  |
|  |  |  |  |  |  |  |  |  |  |  |  |  |  |
|  |  |  |  |  |  |  |  |  |  |  |  |  |  |
|  |  |  |  |  |  |  |  |  |  |  |  |  |  |
|  |  |  |  |  |  |  |  |  |  |  |  |  |  |
|  |  |  |  |  |  |  |  |  |  |  |  |  |  |
|  |  |  |  |  |  |  |  |  |  |  |  |  |  |
|  |  |  |  |  |  |  |  |  |  |  |  |  |  |
|  |  |  |  |  |  |  |  |  |  |  |  |  |  |
|  |  |  |  |  |  |  |  |  |  |  |  |  |  |
|  |  |  |  |  |  |  |  |  |  |  |  |  |  |
|  |  |  |  |  |  |  |  |  |  |  |  |  |  |
|  |  |  |  |  |  |  |  |  |  |  |  |  |  |
|  |  |  |  |  |  |  |  |  |  |  |  |  |  |
|  |  |  |  |  |  |  |  |  |  |  |  |  |  |
|  |  |  |  |  |  |  |  |  |  |  |  |  |  |
|  |  |  |  |  |  |  |  |  |  |  |  |  |  |
|  |  |  |  |  |  |  |  |  |  |  |  |  |  |
|  |  |  |  |  |  |  |  |  |  |  |  |  |  |
|  |  |  |  |  |  |  |  |  |  |  |  |  |  |
|  |  |  |  |  |  |  |  |  |  |  |  |  |  |
|  |  |  |  |  |  |  |  |  |  |  |  |  |  |
|  |  |  |  |  |  |  |  |  |  |  |  |  |  |
|  |  |  |  |  |  |  |  |  |  |  |  |  |  |
|  |  |  |  |  |  |  |  |  |  |  |  |  |  |
|  |  |  |  |  |  |  |  |  |  |  |  |  |  |
|  |  |  |  |  |  |  |  |  |  |  |  |  |  |
|  |  |  |  |  |  |  |  |  |  |  |  |  |  |

Figure 1: Estimated Down syndrome (DS) maternal age relationship after the age of 30 calculated using the combined data of Hecht and Hook (1996) and the UK National Down Syndrome Cytogenetic Registry (Morris et al. 2002). Total = 11,564 cases. Detailed data in online resources Table 1.

Figure 2. Changing proportion of mothers ≥35, 1950-2010 by WHO region. Data from WPP 2012 revision. AFR: African, AMR: American, EMR: Eastern Mediterranean, EUR: European, SEAR: South-East Asian, WPR: Western Pacific Region, W.Europe: Western Europe

Table 2. Average Whipple’s index by WHO region, sex and urban/rural residence in the most recent year recorded in UNDY Special Census Topics 2005. 85% of countries, rates between 1990 and 2003. Index scores range between 100 and 500, a high score (generally above 125) indicates lower quality data.

| WHO region | Urban population | | | Rural population | | |
| --- | --- | --- | --- | --- | --- | --- |
|  | Both sexes | Male | Female | Both sexes | Male | Female |
| AFR | 164 | 157 | 170 | 180 | 171 | 187 |
| AMR | 106 | 105 | 106 | 110 | 111 | 110 |
| EMR | 126 | 123 | 129 | 139 | 138 | 141 |
| EUR | 103 | 102 | 103 | 104 | 103 | 104 |
| SEAR | 236 | 232 | 241 | 276 | 281 | 272 |
| WPR | 101 | 102 | 101 | 102 | 102 | 102 |
| World | 146 | 145 | 148 | 161 | 161 | 160 |
| W Europe | 101 | 101 | 101 | 100 | 100 | 100 |

Figure 3. United Kingdom. Results of two methods of estimating Down syndrome live birth prevalence 1950-2015: Whipple's index (both sexes) 98.0 (1991). The two calculations produce the same result because the equation is based on data for populations with accurate age reporting.

Figure 4. Nigeria. Results of two methods of estimating Down syndrome live birth prevalence 1950-2015: Whipple's index (both sexes) 293.5 (1991) is among the highest given for any country.

Figure 5. Iran. Results of two methods of estimating Down syndrome live birth prevalence 1950-2015. Whipple's index (both sexes) 128.7 (1991). The convergence of the results reflects increasing improving age reporting in the population over time.
